# Hepatic tuberculosis: a multimodality imaging review

**DOI:** 10.1007/s13244-015-0440-y

**Published:** 2015-10-24

**Authors:** Chandan Kakkar, Ashwin M. Polnaya, Prakashini Koteshwara, S. Smiti, K. V. Rajagopal, Ankur Arora

**Affiliations:** Department of Radiodiagnosis and Imaging, Dayanand Medical College and Hospital, Ludhiana, India; Department of Radiodiagnosis and Imaging, Tata Memorial Hospital, Parel, Mumbai, India; Department of Radiodiagnosis and Imaging, Kasturba Medical College and Hospital, Manipal, India; Department of Radiology, Institute of Liver and Biliary Sciences, New Delhi, India

**Keywords:** CT, Hepatic tuberculosis, Miliary, Nodular, Tubercular cholangitis, MRI

## Abstract

**Objectives:**

We aim to illustrate the multimodal imaging spectrum of hepatic involvement in tuberculosis (TB). Whilst disseminated tuberculosis on imaging typically manifests as multiple small nodular lesions scattered in the liver parenchyma, isolated hepatic tuberculosis remains a rare and intriguing entity.

**Methods:**

Indubitably, imaging is the mainstay for detection of tubercular hepatic lesions which display a broad spectrum of imaging manifestations on different modalities. While sonography and computed tomography (CT) findings have been described in some detail, there is a paucity of literature on magnetic resonance imaging (MRI) features. Due to a significant overlap with other commoner and similar appearing hepatic lesions, hepatic tuberculosis is often either misdiagnosed or labelled as indeterminate lesions. This article is a compendium of cases highlighting the spectrum of imaging patterns that can be encountered in patients with isolated primary hepatic tuberculosis as well as disseminated (secondary) disease. Rare patterns of primary disease such as tubercular cholangitis, hypervascular liver masses, and those with vascular complications are also illustrated and discussed.

**Conclusions:**

Imaging plays a valuable role in the detection of tubercular hepatic lesions. Also, imaging can be helpful in their characterisation and for assessing associated complications.

***Teaching points*:**

• *Hepatic TB has myriad imaging manifestations and is often confounded with neoplastic lesions.*

• *Imaging patterns include miliary TB, macronodular TB, serohepatic TB and tubercular cholangitis.*

• *Concurrent splenic, nodal or pulmonary involvements are helpful pointers towards the diagnosis.*

• *Miliary calcifications along the bile ducts are characteristic of tubercular cholangitis.*

• *Histological/microbiological confirmation is often necessary to confirm the diagnosis.*

## Introduction

Tubercular infection constitutes one of the foremost causes of death and morbidity across the world, more so in the tropical region. With the emergence of HIV and AIDS, the disease that was thought to be endemic to emerging nations has become pandemic in nature [1–3]. Approximately 15% of people infected with HIV are co-infected with tuberculosis, pulmonary as well as extra-pulmonary in distribution, thus making the disease one of the leading causes of death in this population [4, 5].

Tubercular involvement of the liver is more commonly a part of disseminated disease wherein the hepatic parenchyma shows a diffuse pattern of involvement in the form of multiple small-sized miliary nodules. In contrast, isolated hepatic tuberculosis is seldom encountered in clinical practice with only a few sporadic cases and short series available in the current literature [6–9].

Although there is no standard classification system available for hepatic tuberculosis, Levine [10] classified hepatic involvement in tuberculosis into five patterns: miliary tuberculosis, concomitant hepatic and pulmonary disease, primary (isolated) hepatic tuberculosis, tubercular hepatic abscess, and tubercular cholangitis. Also, there is a pathological classification with radiological correlation wherein Yu et al. [9] classified the disease into three forms: parenchymal type (which is further subclassified into micronodular and macronodular patterns), serohepatic disease and tubercular cholangitis.

## Clinical features

Hepatobiliary tuberculosis most commonly affects people in the 11– to 50-year-old age group with the peak incidence of the disease reported in the second decade of life [11]. The disease has a 2:1 male preponderance. Isolated hepatic tuberculosis is however more common in the fourth to sixth decades of life [7, 12, 13].

Generally, the disease remains silent and is often incidentally detected while the patient is being evaluated for a mostly non-specific symptomatology. It may however present in the form of abdominal pain or organomegaly which may or may not be associated with clinical jaundice. Jaundice if present is usually a consequence of extrahepatic biliary obstruction secondary to attendant periportal lymphadenopathy [14, 15].

Laboratory analysis may reveal altered liver function tests in the form of elevated hepatic enzymes. The elevation of alkaline phosphatase can be seen in jaundiced as well as non-jaundiced patients. In the event of isolated elevation of the alkaline phosphatase the possibility of tubercular hepatic parenchymal involvement must be excluded [11, 13].

## Imaging features

The imaging manifestation of the tubercular hepatic disease can be wide ranging but can be broadly categorised into miliary pattern, nodular tuberculosis with serohepatic variant and tubercular cholangitis. As imaging pattern is largely non-specific, a histopathological or bacteriological confirmation is often required.

## Miliary tuberculosis

**Fig. 1:**
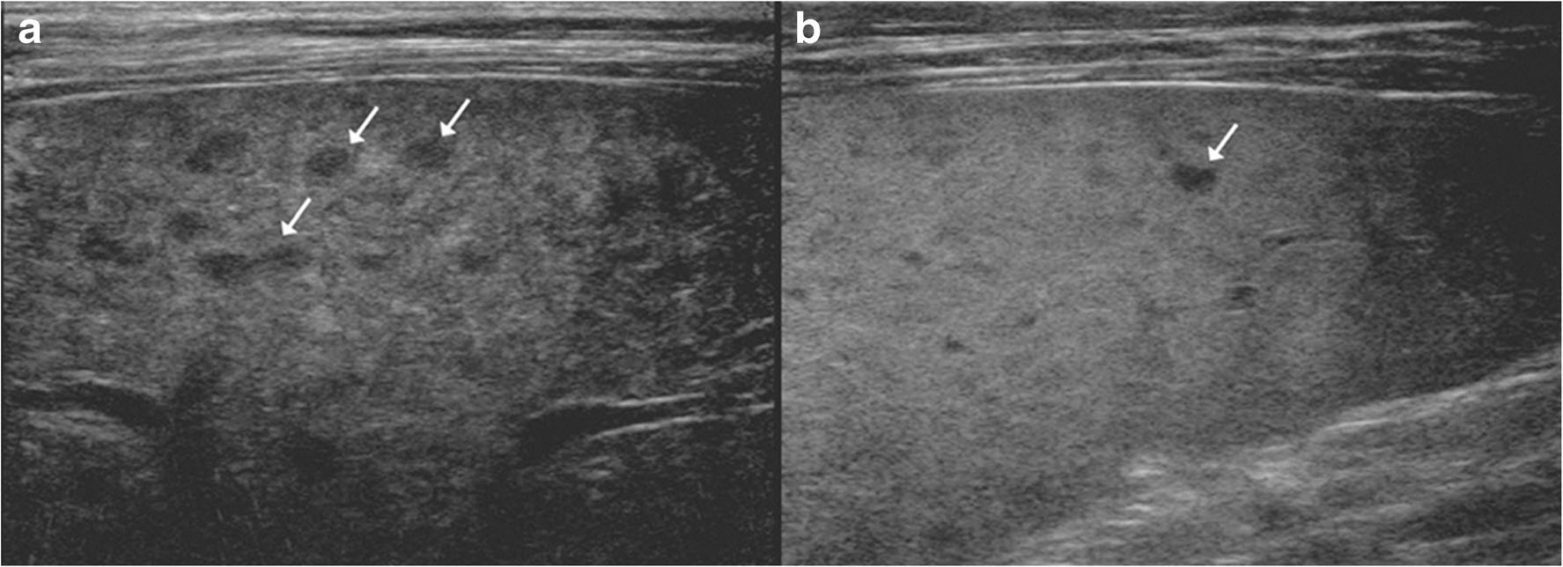
A 46-year-old man with disseminated tuberculosis and elevated liver enzymes. **a**, **b** Ultrasound images show multiple hypoechoic lesions in the liver and spleen (*arrows*) in keeping with granulomas

**Fig. 2:**
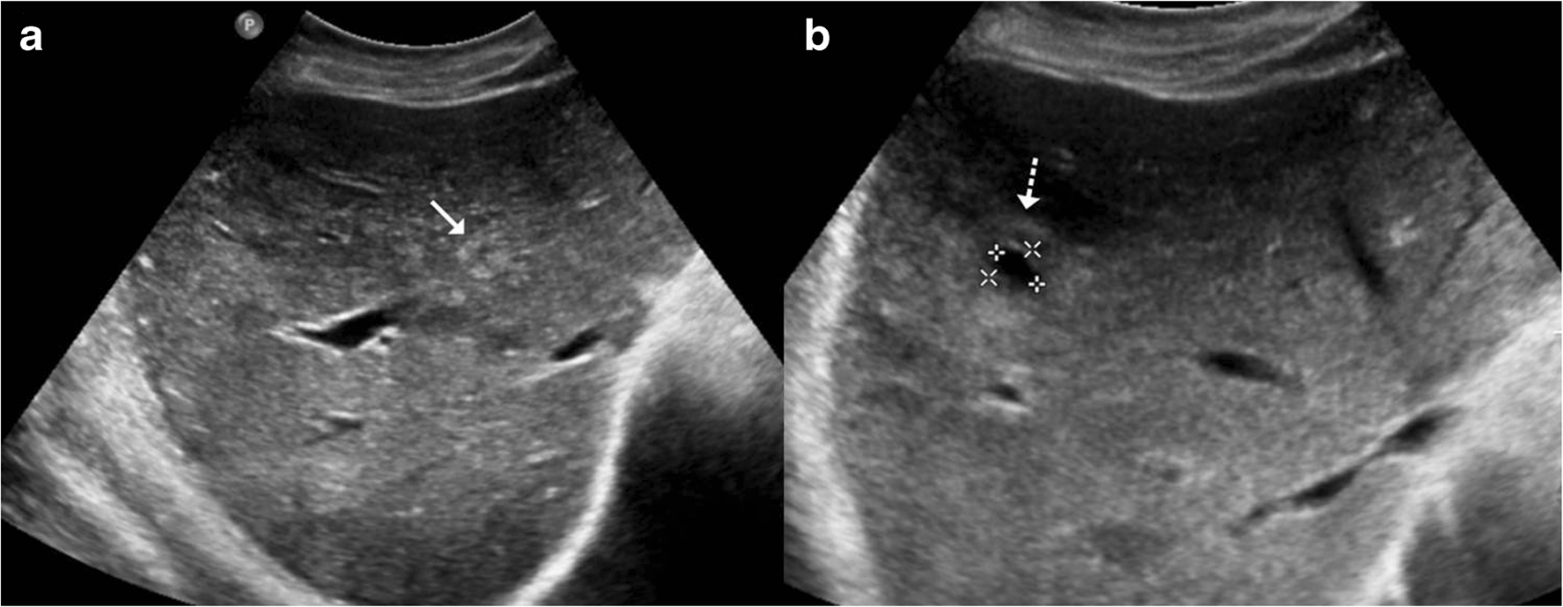
**a** Hepatic tuberculosis manifesting as hyperechoic liver lesions on ultrasonography (*arrow*). **b** One of the larger lesions shows central caseation and necrosis (*dotted arrow*)

It is the most common pattern of hepatic involvement by tuberculosis. Miliary pattern is usually associated with disseminated disease. Accordingly, concurrent involvement of the spleen and other abdominal organs is not uncommon [16]. The miliary hepatic lesions are multiple, generally less than 2 cm in size and tend to be randomly distributed across the entire hepatic parenchyma. Sonographically, these lesions appear hypoechoic to isoechoic relative to the background parenchyma (Fig. 1); however, in rare instances a hyperechoic pattern may be demonstrated (Fig. 2)[6, 8, 12, 17–19].

**Fig. 3:**
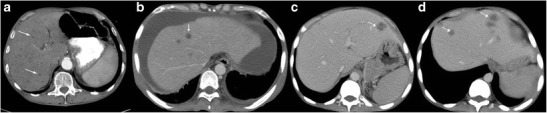
**a** Axial CT image of a 45-year-old woman with disseminated tuberculosis, showing multiple tiny hypodense lesions (*thin arrows*) in both lobes of the liver. **b** Disseminated disease in a 25-year-old man with multiple low attenuation cystic appearing lesions (*short arrows*) in the right lobe of the liver and associated ascites. **c**, **d** Contrast CT in a middle-aged woman with fever and disseminated tuberculosis exhibiting multiple hypodense lesions with subtle peripheral enhancement (*dashed arrows*)

**Fig. 4:**
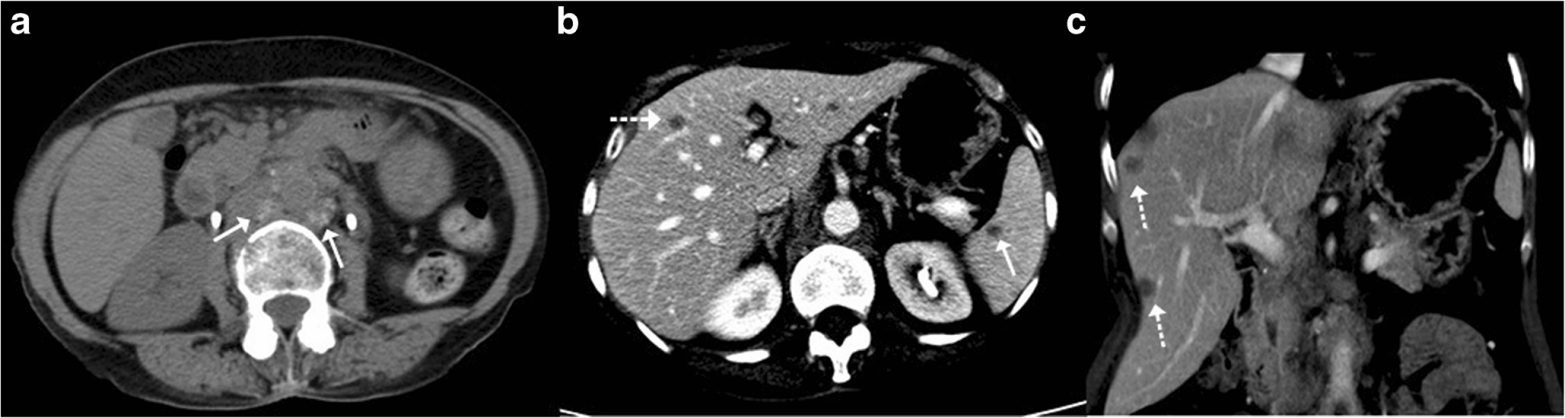
**a** Unenhanced CT of a 65-year-old woman with a history of weight loss showing retroperitoneal nodes with calcification (*arrows*). **b**, **c** Post-contrast CT images showing small hypodense lesions in both lobes of the liver (*dashed arrows*). Biopsy from the retroperitoneal nodes revealed metastases from mucinous adenocarcinoma

**Fig. 5:**
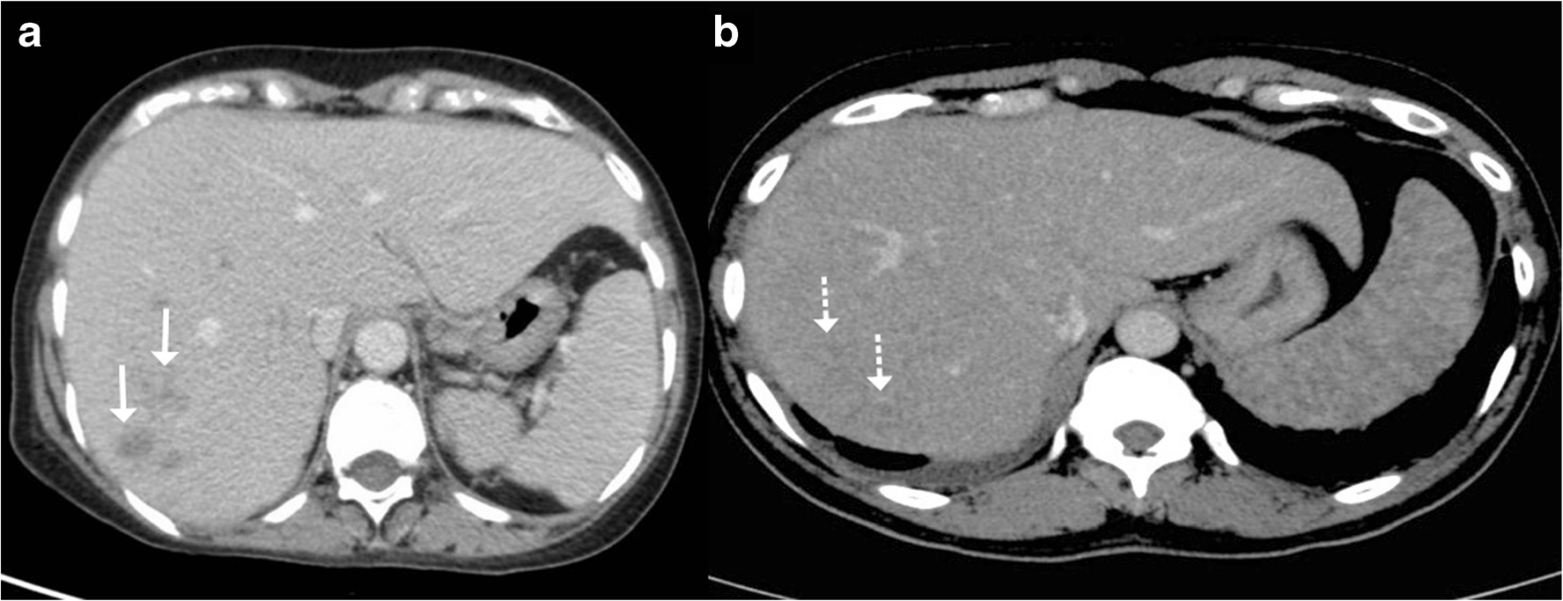
**a** Axial contrast-enhanced CT of a 55-year-old woman with fever and weight loss exhibits multiple hypodense lesions (*arrows*) in the right lobe of the liver. Biopsy from the lesions revealed null cell lymphoma. **b** CT images of a 40-year-old man with known sarcoidosis shows subtle hypodense hepatic lesions (*dashed arrows*) with heterogeneous appearance of the spleen

On computed tomography (CT), miliary lesions appear as microabscesses in the form of multiple small foci with a low attenuation ranging between 30 to 40 HU. The lesions appear nodular or cystic and may exhibit minimal peripheral enhancement following intravenous contrast administration (Fig. 3). This pattern can make it difficult to distinguish them from metastases (Fig. 4), lymphoma (Fig. 5a) or other forms of granulomatous diseases (Fig. 5b) [20].

**Fig. 6:**
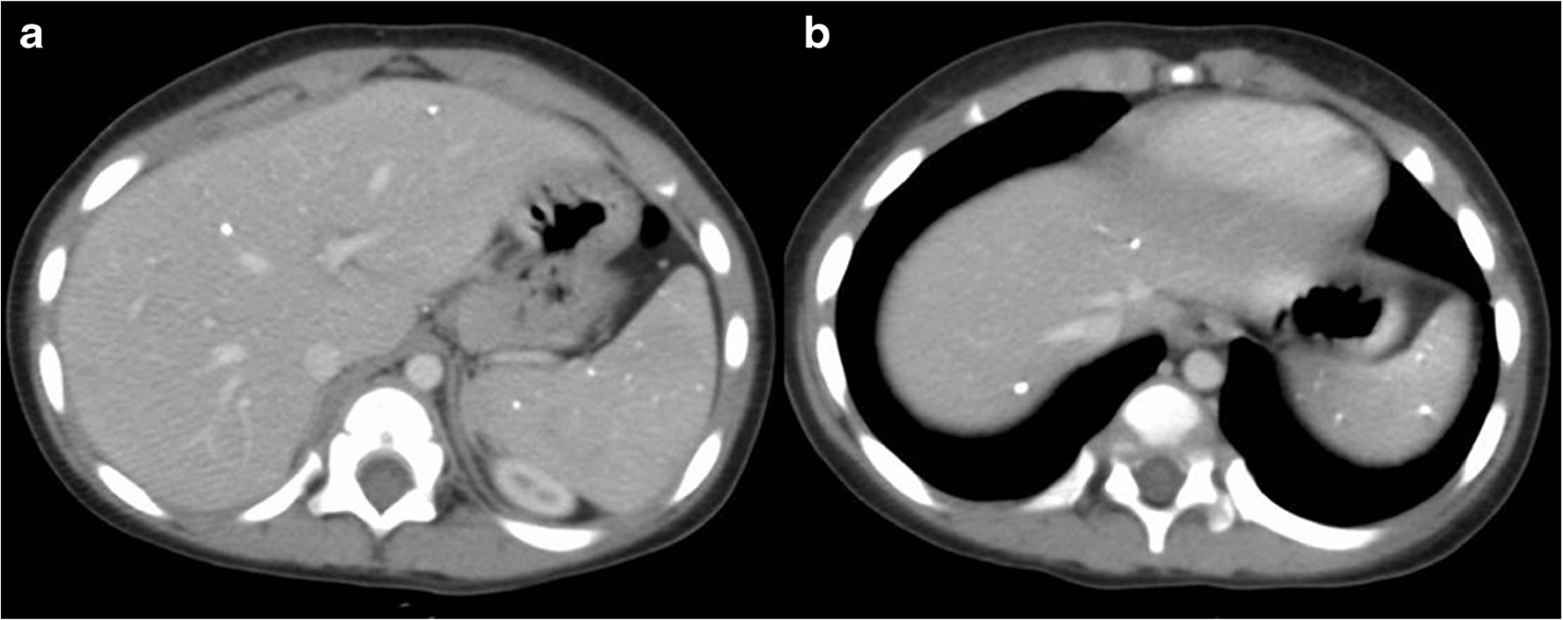
A middle-aged woman with a history of treated tuberculosis. **a**, **b** Axial contrast-enhanced CT images reveal multiple calcified lesions in the liver and spleen in keeping with calcified granulomas

Calcification is not uncommon in long-standing chronic disease. In fact, these lesions are better discernible and more frequently discovered at this stage, where they manifest as small nodular foci of calcifications (Fig. 6) [9].

## Macronodular tuberculosis

Tubercular hepatic lesions that are more than 2 cm in size are referred to as macronodular or pseudotumoral tuberculosis. This form of hepatic tuberculosis is rare compared to the miliary variant and frequently manifests as solitary or multiple variable-sized hepatic masses. Often it may be difficult to distinguish these lesions from the more common neoplastic and other infective lesions. Depending on the stage of the hepatic granuloma, the imaging appearances can be quiet variable [6–10, 21].

**Fig. 7:**
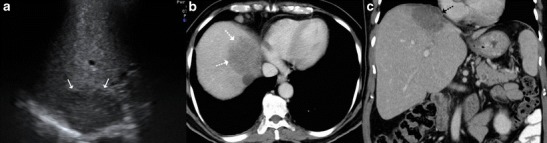
A 78-year-old man with weight loss and anorexia. **a** Ultrasound shows a well-defined hypoechoic lesion in the right lobe extending up to capsular surface (*arrows*). **b**, **c** Axial and coronal contrast-enhanced CT images show minimally enhancing subcapsular lesion in the right lobe (*white dashed arrow*) with associated capsular thickening (*black dashed arrow*)

**Fig. 8:**
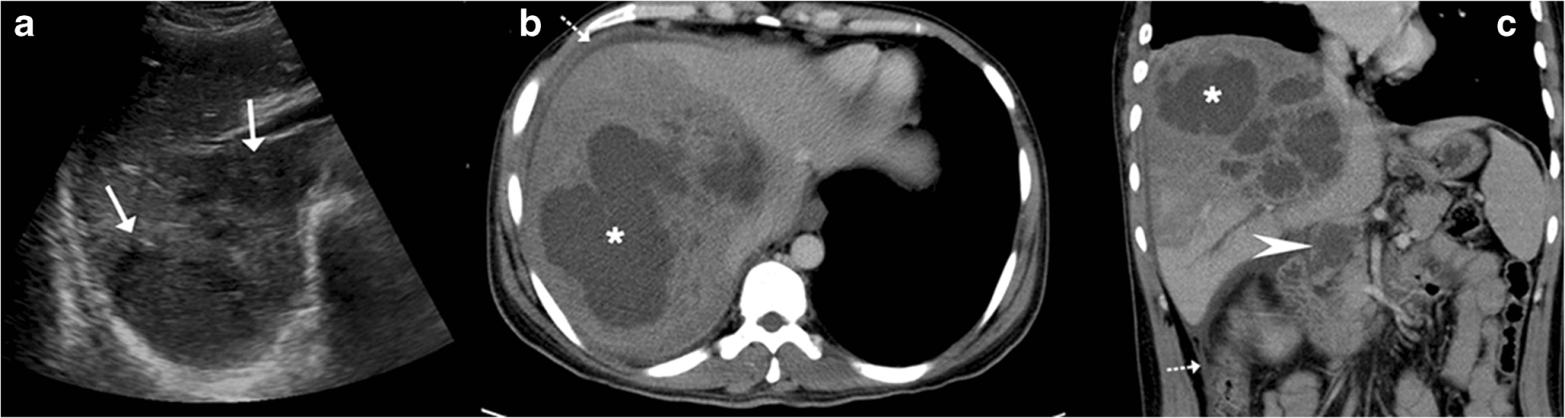
A 20-year-old man with fever. **a** Ultrasound shows a large, ill-defined heterogeneous lesion in the right lobe with relatively hypoechoic areas (*arrows*) suggestive of liquefaction. **b**, **c** Axial CT image in the portovenous phase shows the multiseptated peripherally enhancing lesion with septal enhancement and central necrosis (*asterisk*). Additionally, peritoneal thickening (*dotted arrow*) and infra-hepatic necrotic lymph nodal mass is noted (*arrowhead*). Patient underwent pigtail catheter drainage and culture and polymerase chain reaction (PCR) analysis revealed acid fast bacilli

**Fig. 9:**
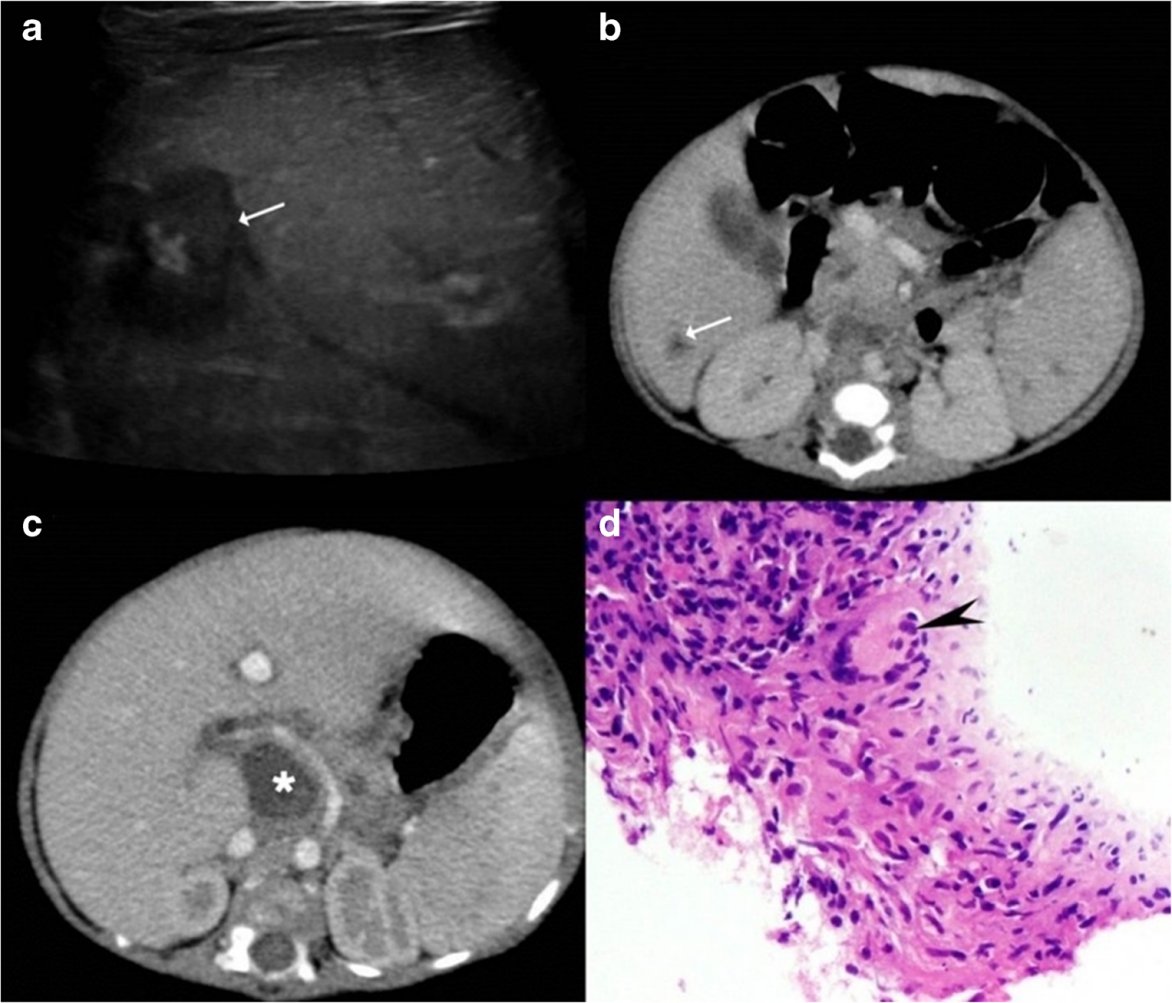
A 3-month-old child with failure to thrive. **a** Ultrasound shows a hypoechoic lesion in the right lobe with central hyperechoic area. **b** Portal venous phase CT reveals a small hypodense lesion in the right lobe with concurrent splenic lesions. **c** A large necrotic lymph node (*asterisk*) is also identified. **d** Histopathology from the hepatic lesion revealed a giant cell granuloma. This was a case of vertical transmission as mother was an active case of tuberculosis during pregnancy and delivery

**Fig. 10:**
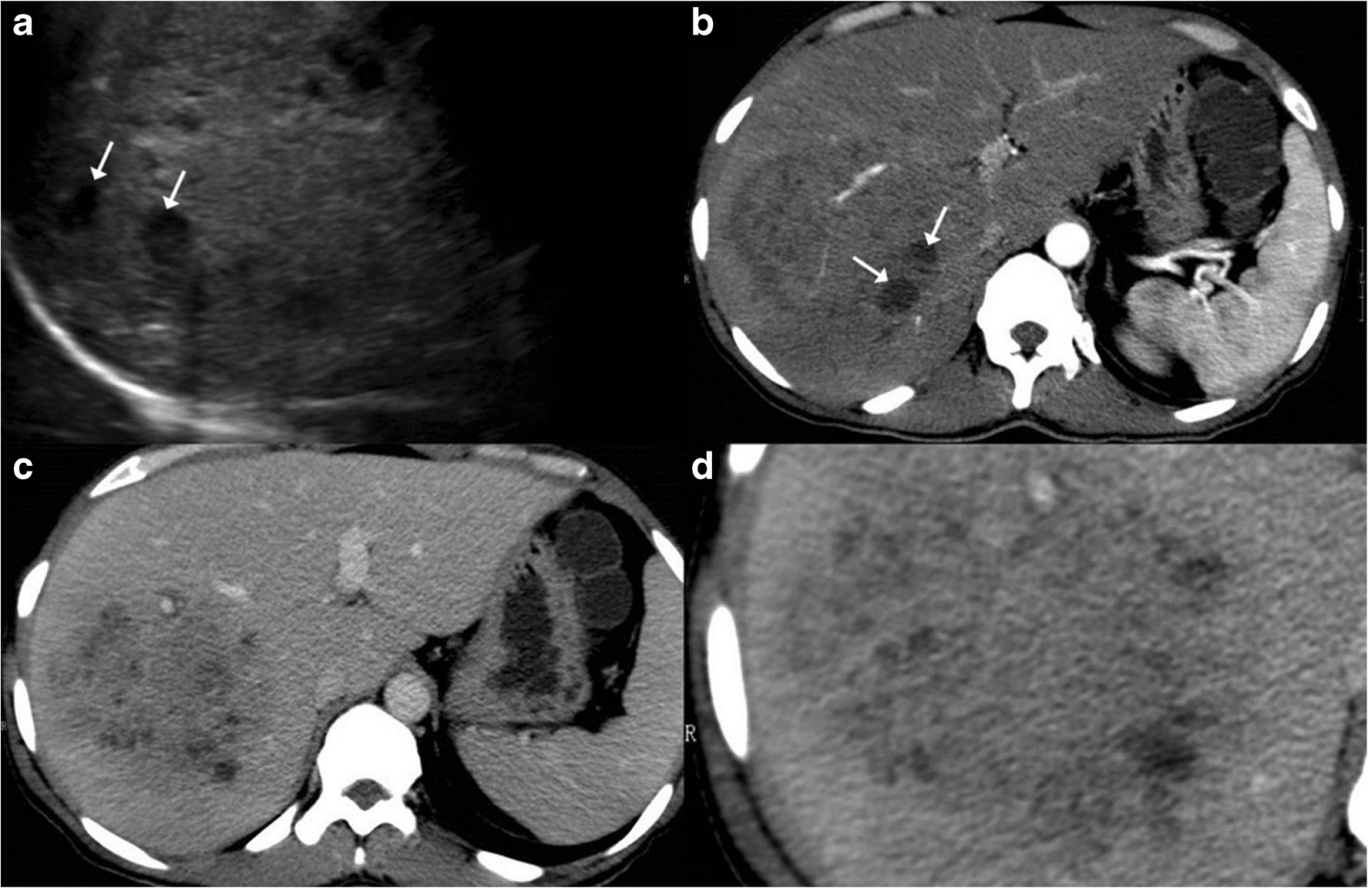
A 27-year-old man with fever, anorexia and isolated hepatic involvement. **a** Ultrasound shows an ill-defined heterogeneous area in the right lobe with a few well-defined hypoechoic areas (*arrows*) suggestive of liquefaction. **b**, **c** Arterial and portal venous phase reveals a well-circumscribed enhancing mass composed of multiple tiny abscesses. Vessels can be seen coursing through the lesion without being attenuated or infiltrated. **d** Magnified view shows tiny abscesses in a large complex mass giving a ‘cluster’ appearance

Sonographically, these lesions can range from being heterogeneously hypoechoic (Fig. 7a), mixed hypoechoic to hyperechoic with anechoic areas of breakdown (Fig. 8a), predominantly hyperechoic to rarely a hypoechoic lesion with a hyperechoic centre (Fig. 9a). These lesions tend to display ill-defined borders especially when multiple small hypoechoic lesions coalesce to form a large mass (Fig. 10a), but at times the lesions can show a strikingly very well-defined wall [6, 10, 12, 18, 19, 22].

**Fig. 11:**
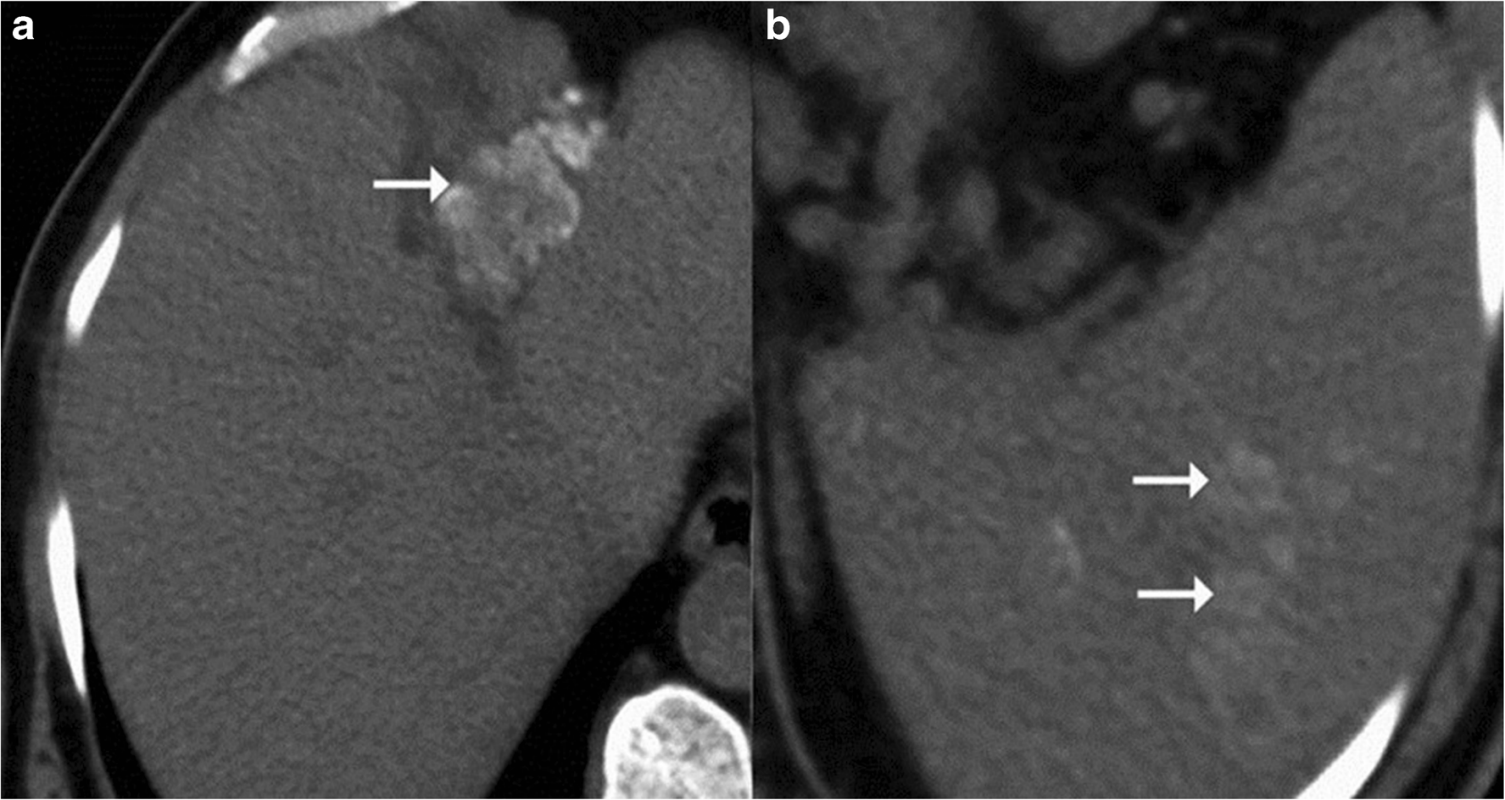
**a**, **b** Plain CT images shows lesion with dense nodular calcification in the left lobe up to the interlobar fissure with concomitant multiple calcified lesions in spleen (*arrows*)

**Fig. 12:**
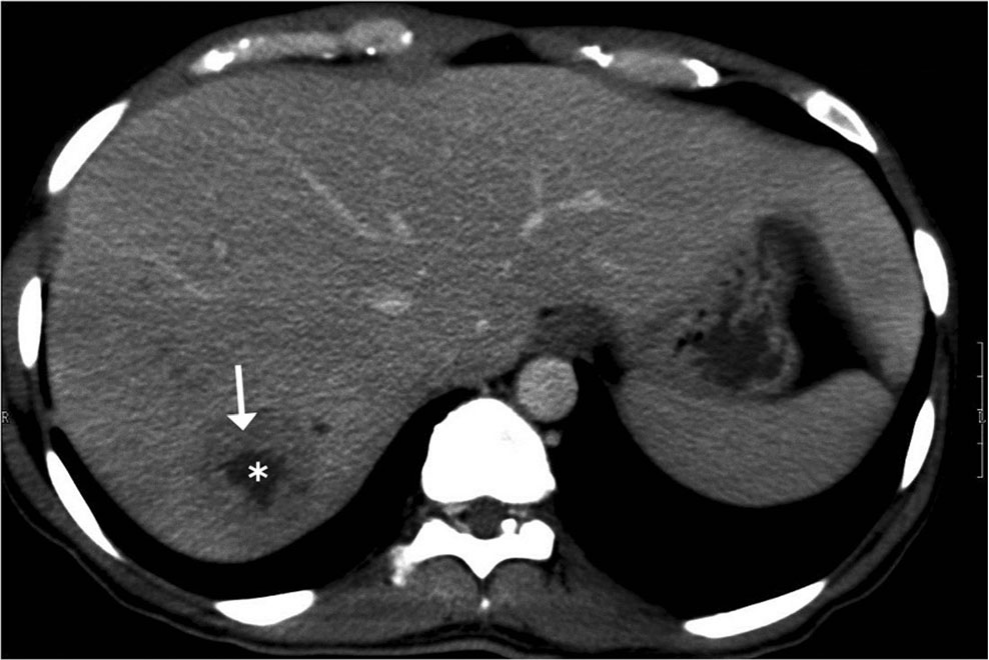
A case of nodular isolated hepatic tuberculosis. Axial contrast-enhanced CT shows a well-defined lesion in the right lobe with thick enhancing rim (*arrow*) and central area of necrosis (*asterisk*)

CT imaging features of macronodular granuloma of the liver depends on the stage of the disease. Non-caseating granulomas appear hypodense on the unenhanced study and usually display no or minimal peripheral rim enhancement following intravenous contrast administration (Figs. 7b, c and 9b, d). Understandably, such a lesion if present in isolation can pose a diagnostic dilemma making it practically impossible to differentiate it from hepatic metastasis or other primary tumours. The lesion can in addition show punctuate or chunky internal calcifications on CT (Fig. 11) [9, 23, 24]. Lesions with frank caseous necrosis result in tubercular abscesses whose imaging appearances vary depending upon the degree of internal liquefaction. The liquefaction can be multifocal (Fig. 10b, d) or central (Fig. 12) [6, 9, 10]. Similar to pyogenic abscesses, tubercular abscesses can at times exhibit a “honeycomb” appearance with multiple enhancing septations and intermixed areas of necrosis (Fig. 8b, c) [25]. A conglomeration of these cystic lesions can give rise to the “cluster sign”, which is more often associated with pyogenic or cholangitic abscesses Occasionally, the lesions can display extensive necrosis thus mimicking cysts depicting no discernible peripheral enhancement [9, 13, 22].

**Fig. 13:**
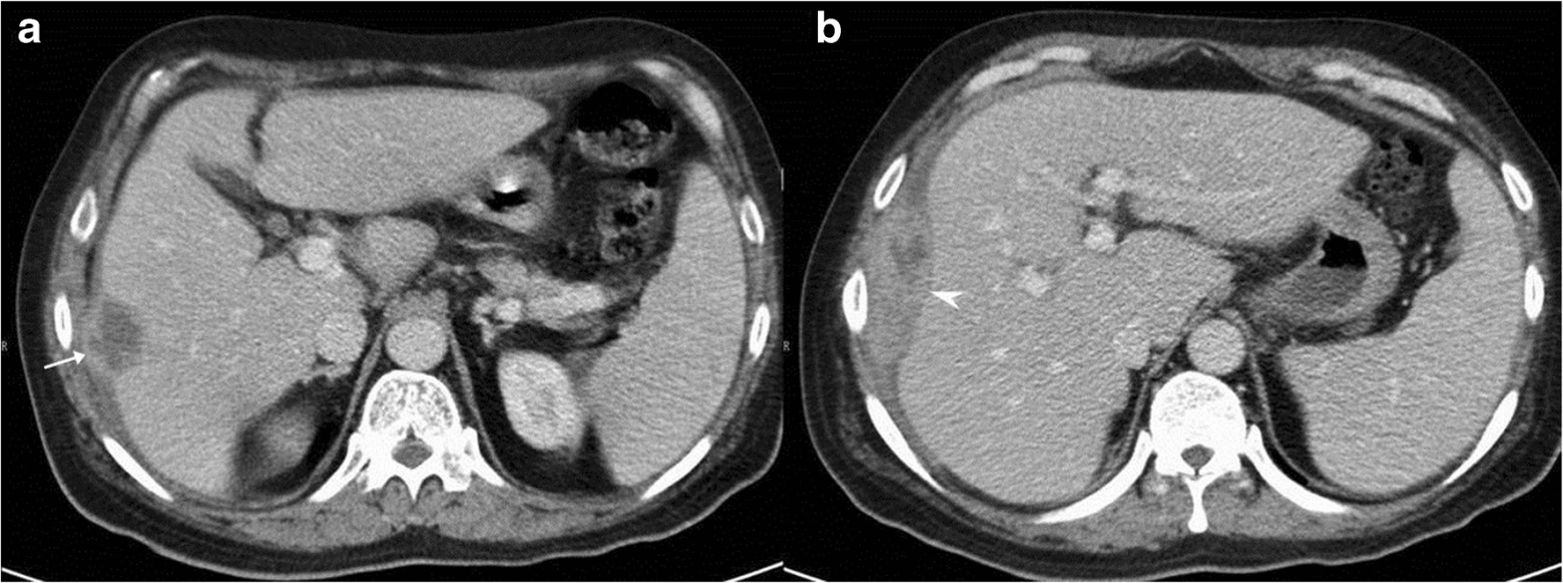
A 45-year-old man with right upper quadrant pain and fever. **a**, **b** Axial portal venous phase CT shows a hypodense lesion in the right lobe causing focal contour bulge and capsular thickening (*arrow*) with an associated organised collection in the perihepatic space (*arrowhead*). This was a patient with isolated hepatic tuberculosis with contained rupture of the tubercular liver abscess

**Fig. 14:**
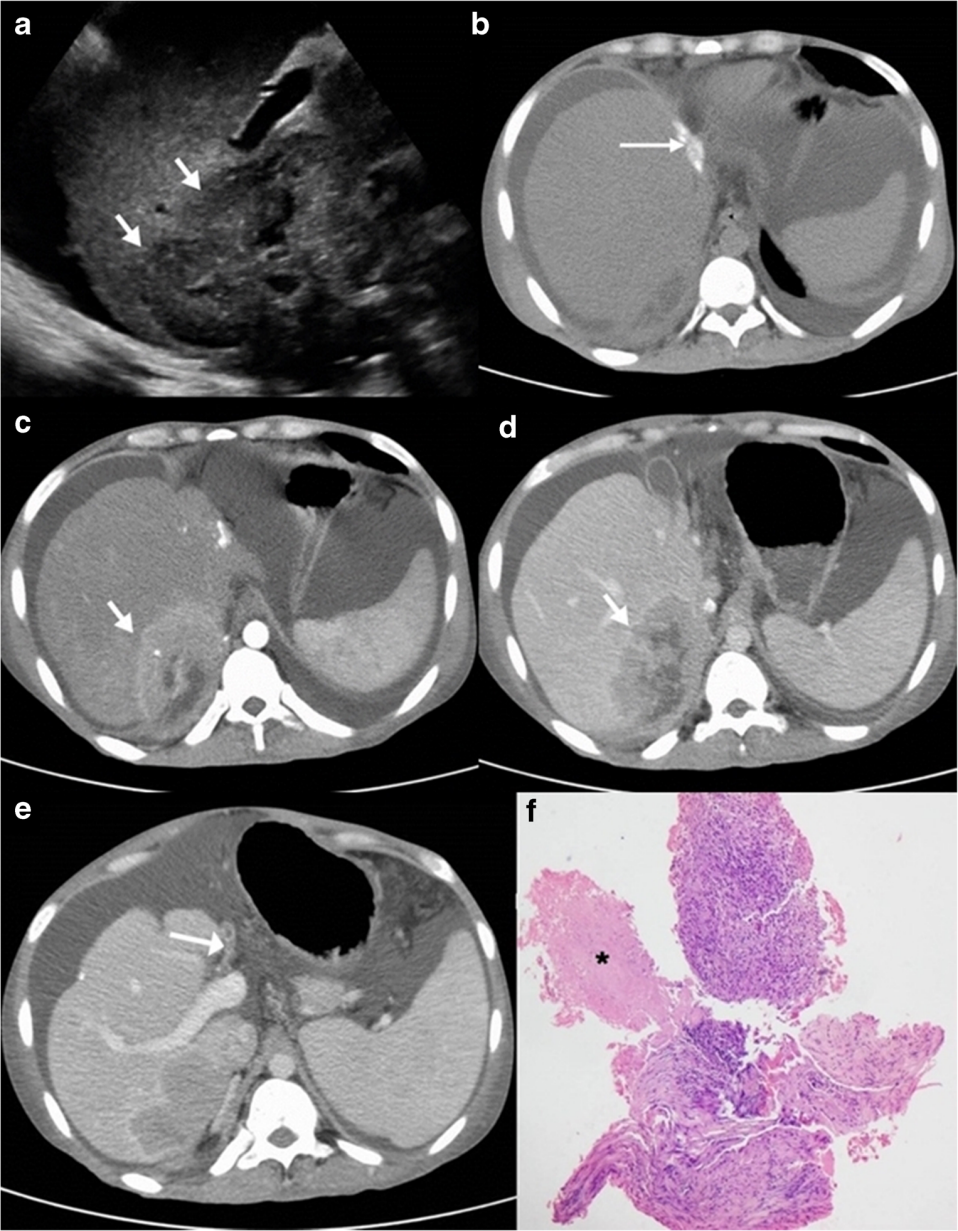
A 22-year-old man with features of hepatic failure. **a** Ultrasound shows a heterogeneously hypoechoic mass (*arrows*) in the right liver lobe. **b** Plain CT image shows dense calcification along the interlobar fissure with non-visualisation of the left lobe. **c**, **d** On arterial images, the lesion depicts arterial enhancement and central necrosis whilst it appears hypodense in the portal venous phase thus simulating a hepatocellular carcinoma. **e** Caudal sections reveal attenuated and thrombosed left portal branch (*arrow*) and normal appearing right and main portal branch. **f** Core biopsy specimen shows few hepatocytes and extensive caseous necrosis (*asterisk*)

Rarely, tubercular liver abscesses can rupture resulting in extra-hepatic complications (Fig. 13) such as perihepatic abscess, infective peritonitis, etc. [26]. Vascular complications like portal vein thrombosis and subsequent portal hypertension have also been reported (Fig. 14) [27].

**Fig. 15:**
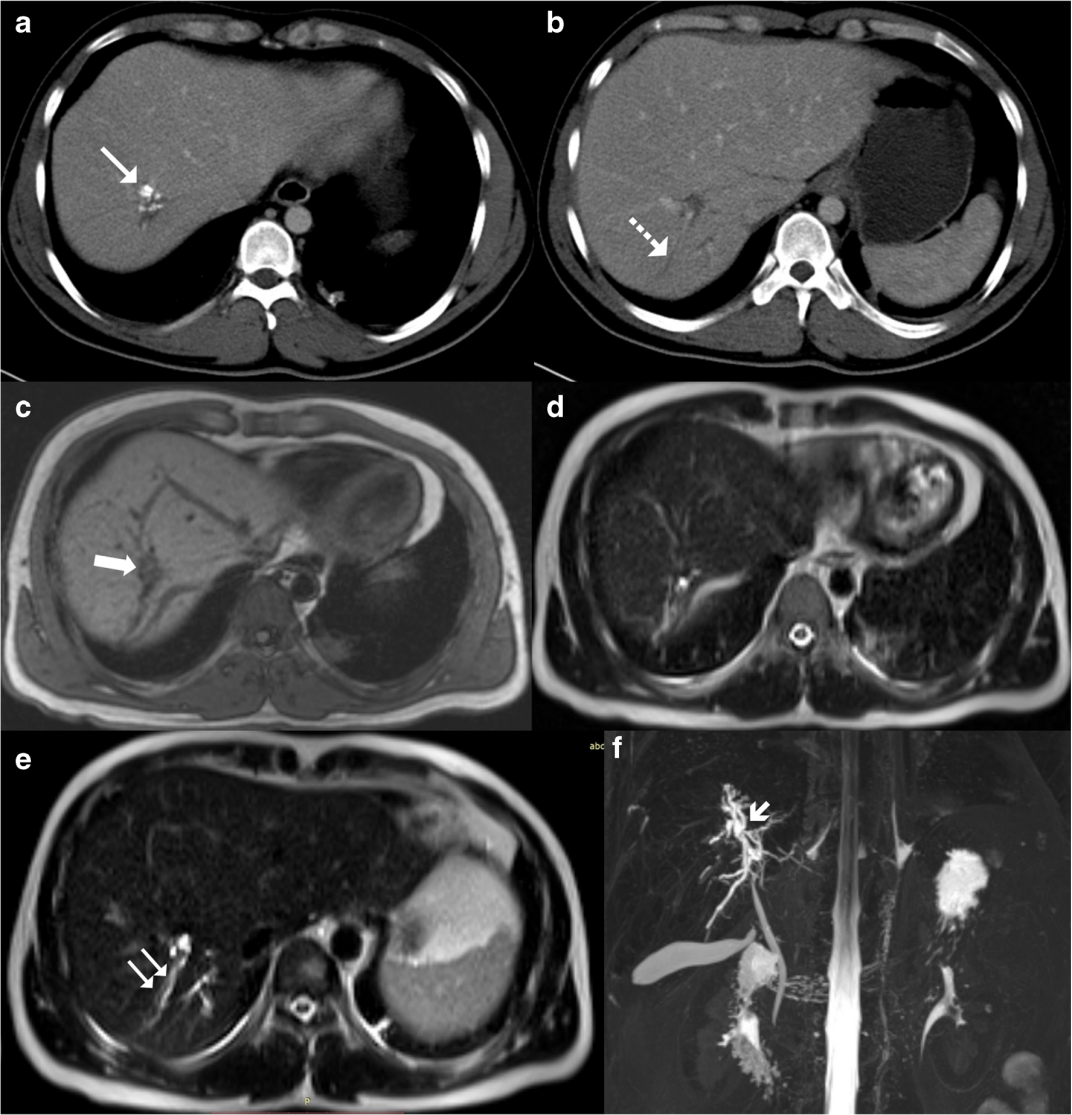
A 35-year-old man previously treated for pulmonary tuberculosis now presenting with elevated alkaline phosphatase. **a**, **b** Axial contrast-enhanced CT reveals chunky calcified nodular lesion in the segment VII of right lobe (*arrow*) with adjacent biliary radical dilatation (*dotted arrow*). **b**, **c** Axial T1- and T2-weighted MRI shows the corresponding lesion to be hypointense on T1 (*thick arrow*) and isointense to liver parenchyma on T2-weighted sequence. **e**, **f** Axial T2-weighted MRI reveals focal biliary radical dilatation (*paired arrows*), which is very well depicted on the MRCP image (*short arrow*)

On magnetic resonance imaging (MRI), macronodular tubercular lesions appear hypointense on T1-weighted images and hypointense, isointense or hyperintense lesions with a peripheral hypointense rim on T2-weighted images depending upon the stage of the disease (Fig. 15a-d). Post contrast, these lesions may show rim or heterogeneous enhancement [8, 9].

## Serohepatic tuberculosis

**Fig. 16:**
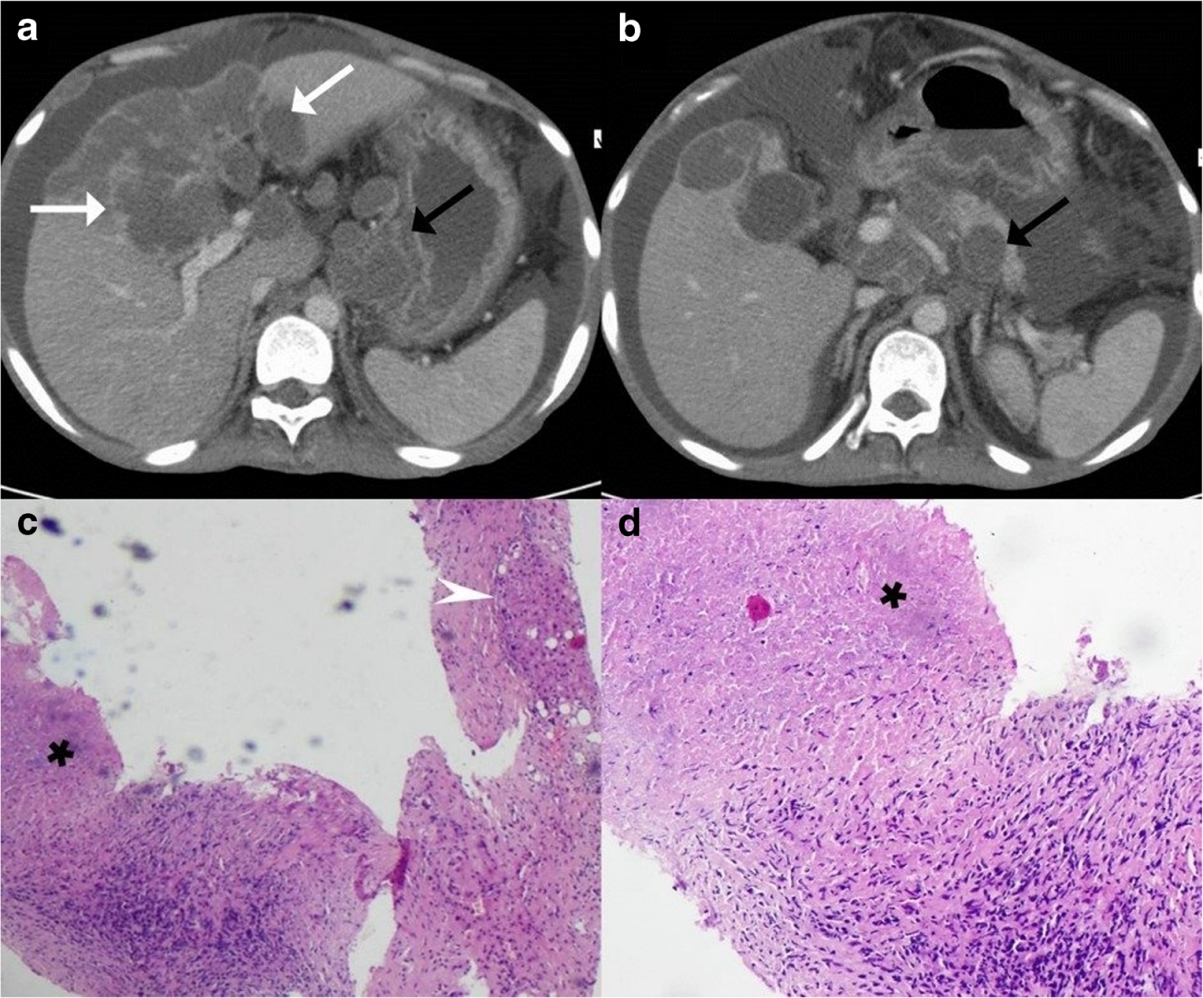
A 53-year-old man with fever and weight loss. **a**, **b** Axial contrast-enhanced CT showing peripherally located conglomerated hypodense lesions (*white arrows*) contiguously extending along the subcapsular plane of the right lobe, caudate lobe and on either side of the falciform ligament. The overlying thickened enhancing capsule simulates ‘sugar coating’. There is associated bulky necrotic gastrohepatic and retroperitoneal adenopathy (*black arrows*). **c**, **d** Low- and high-power miscroscopy reveal focally preserved liver tissue (*arrowhead*) intermixed with areas of caseous necrosis (*asterisk*)

Serohepatic tuberculosis is the rarest variant reported in the literature characterised by predominant involvement of the subserosal plane of the liver i.e. the connective tissue lying beneath the serous coat of liver which is often inseparable from the fibrous Glisson capsule. The imaging features of this subtype include peripherally positioned lesions centred in the subcapsular plane of the liver. The thickened liver capsule and sub-capsule overlying these hypoattenuating lesions simulates a ‘sugar-coating’, an appearance popularly referred to as “frosted liver” (Fig. 16) [9].

## Tubercular cholangitis

**Fig. 17:**
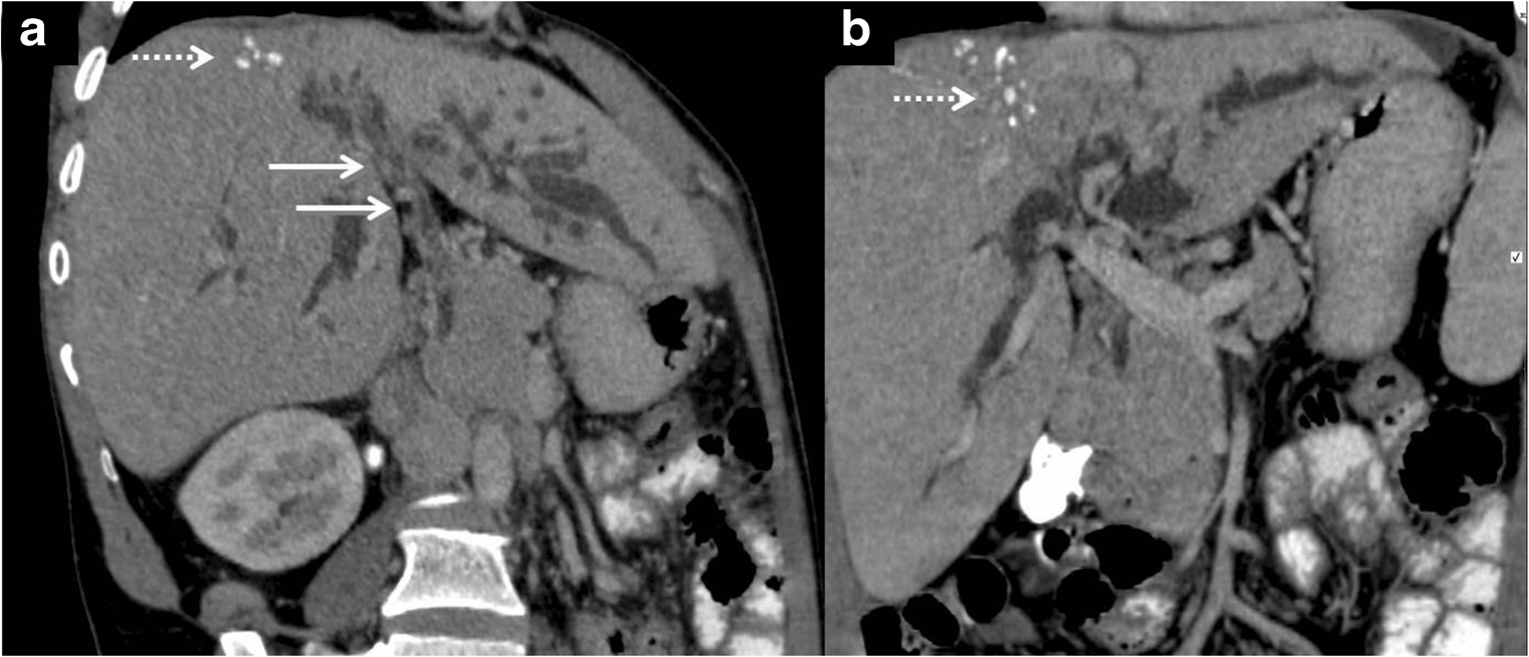
Coronal-oblique and coronal CT images in a patient with tubercular cholangitis displaying thickening and stricture of the extrahepatic common duct (*arrows*) with upstream intrahepatic biliary dilatation. The presence of hepatic calcifications (*dotted arrows*) help exclude cholangiocarcinoma and instead consider tuberculosis in appropriate clinical settings

**Fig. 18:**
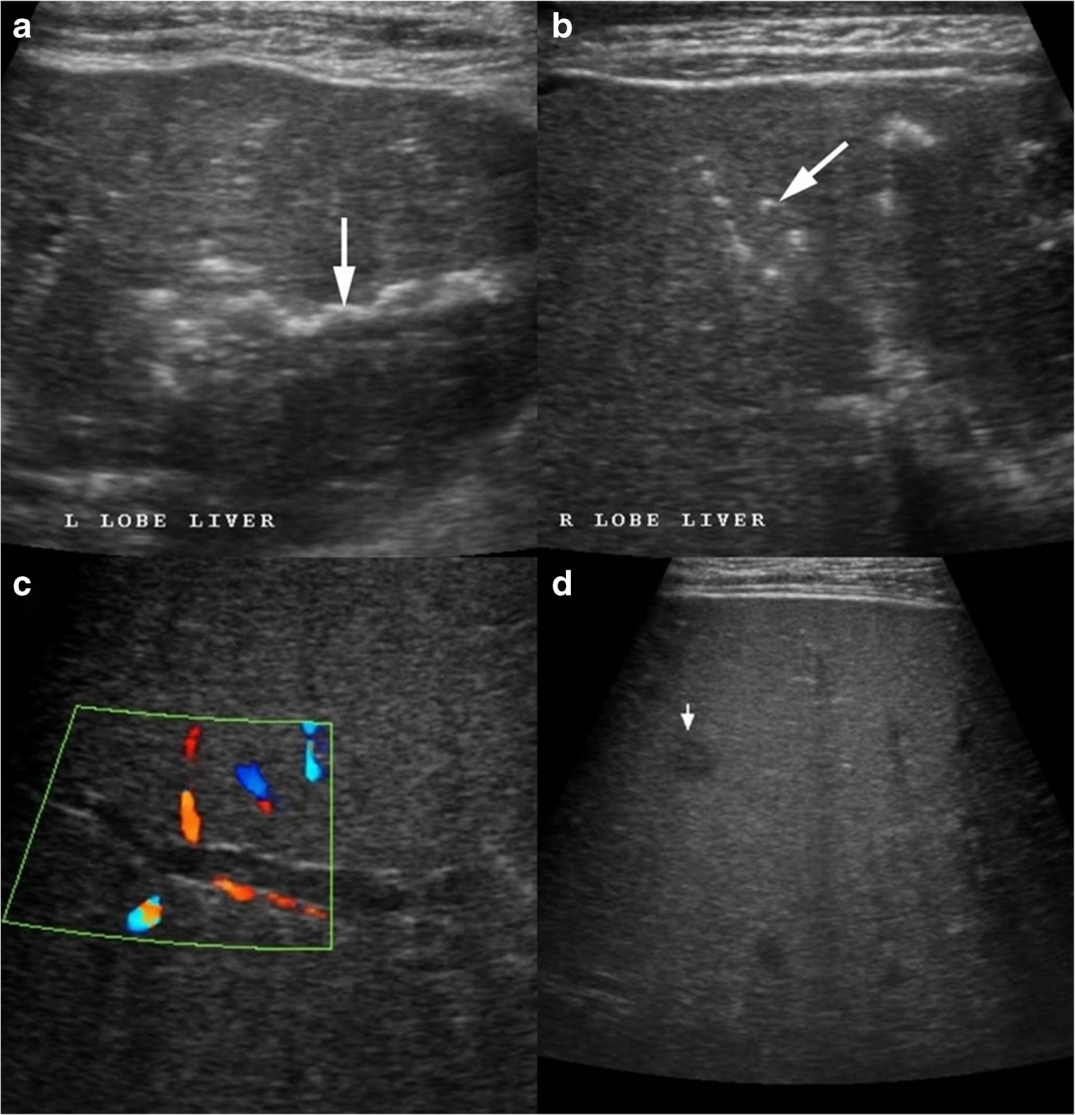
A 28-year-old man a known case of disseminated tuberculosis presenting with jaundice. **a**, **b** Ultrasound shows multiple areas of linear and miliary calcifications (*arrow*) in both lobes of liver. **c** Colour Doppler image shows dilated biliary radical with peripheral wall calcification. **d** Additionally, there is a well-defined small hypoechoic parenchymal lesion likely of a granuloma (*short arrow*)

**Fig. 19:**
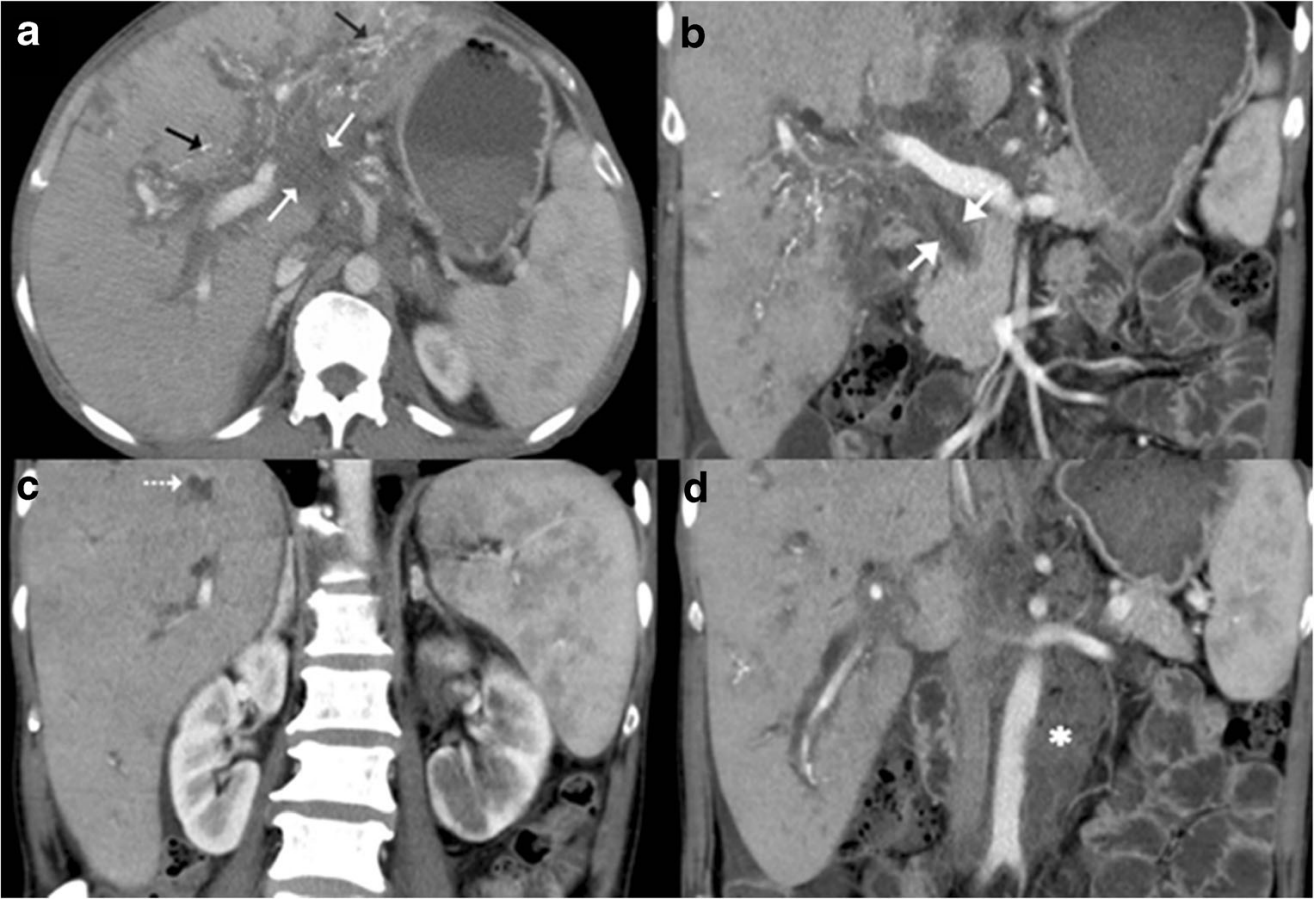
**a**, **b** Contrast-enhanced CT shows an ill-defined mass in the periportal location (*short, thin arrows*) with associated rather marked thickening of the extrahepatic common duct (*short, thick arrow*). Extensive calcifications can be seen along the biliary radicals (*black arrows*). **c** Concurrent hypodense liver lesions either granulomas or cholangitic abscesses (*dotted arrows*). **d** Associated large retroperitoneal nodal mass (*asterisk*) is visualised

**Fig. 20:**
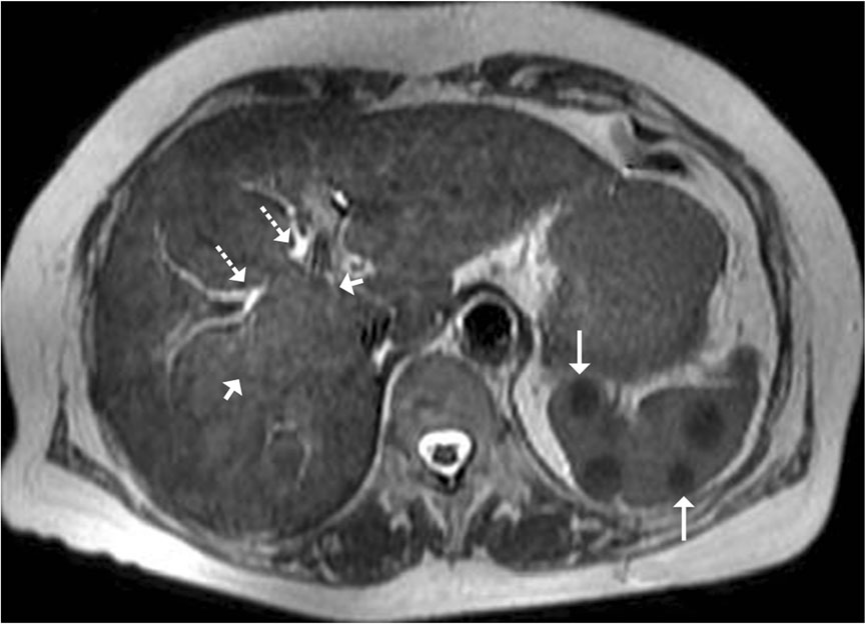
Axial T2-weighted MRI displaying an ill-defined heterogeneous area (*short arrows*) in the right lobe of liver corresponding to granulomatous infiltration, which extends into the periportal location, resulting in central biliary radical dilatation (*dotted arrows*) with concurrent multiple hypointense splenic nodules (*arrows*) in a patient with hepatobiliary tuberculosis

Tubercular cholangitis can either be due to primary involvement of the biliary tree or secondary involvement of the biliary tree due to compression by enlarged periportal nodes and/or hepatic granulomas. Biliary tree involvement can be variable i.e. may involve either the (small) intrahepatic biliary radicles or the larger (juxtahilar or extrahepatic) bile ducts. Imaging findings can vary from bile duct thickening, biliary dilatation and strictures to frank obstructive biliopathy (Fig 17). Allied findings like hepatic calcifications or tuberculomas may be seen. Miliary calcifications along the bile ducts which can be visualised on sonography as well as CT have been described as one of the characteristic features of tubercular cholangitis (Figs. 18 and 19). In the absence of calcifications it can be challenging to differentiate hepatobiliary tuberculosis from cholangiocarcinoma or sclerosing cholangitis. Concomitant disease elsewhere such as nodal or pulmonary involvement can be a useful clue to narrow down the list of differentials (Fig. 20) [9, 28].

**Fig. 21:**
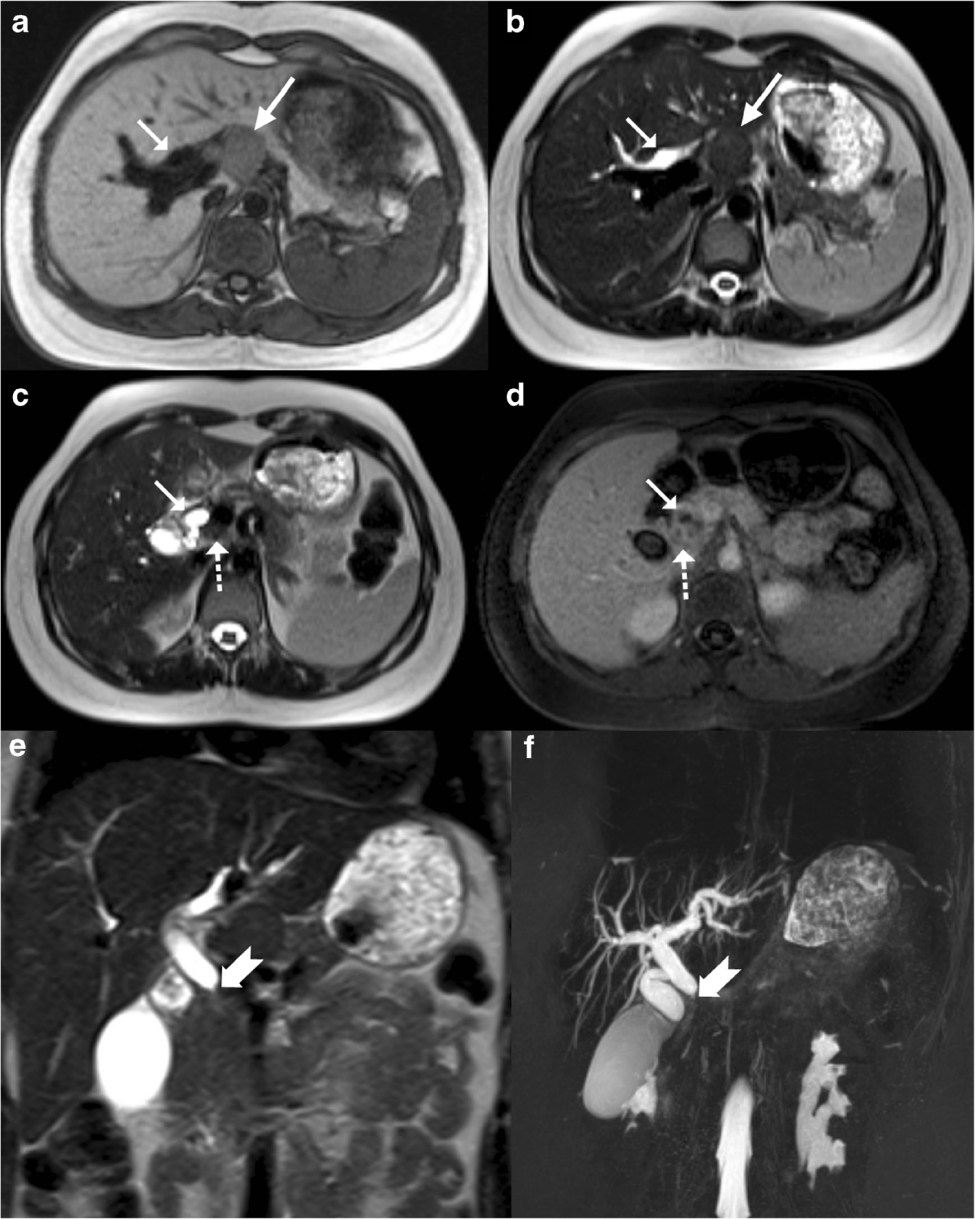
A 21-year-old young woman a known case of disseminated tuberculosis with obstructive jaundice. **a**, **b** Axial T1- and T2-weighted MRI display dilated biliary tree (*arrow*) with a non-necrotic nodal mass at the hepatic hilum (*long arrow*). **c** Axial T2-weighted MRI reveals a smaller node (*dotted arrow*) along the central bile duct (CBD; *arrow*), which is dilated. **d** Post-contrast T1-weighted image shows mild enhancement in the node as well as along the walls of the CBD. **e**, **f** Coronal T2-weighted MRI and thick-slab 3D MRCP beautifully delineates the tight narrowing of the mid extrahepatic common duct in this patient

MRI and magnetic resonance cholangiopancreaticography (MRCP), endoscopic ultrasound, and endoscopic retrograde cholangiopancreaticography (ERCP) also play an important role in the diagnosis and management of patients with biliary tuberculosis. MRCP depicts the biliary anatomy and pathology not only non-invasively (Figs. 15e, f and 21) but also to a better extent when compared to sonography and CT. Endoscopic techniques are invaluable for tissue samplings as well as the placement of stents in obstructed systems [28].

## Conclusions

Hepatic tuberculosis has many faces and the imaging manifestation can show a considerable overlap with other relatively more frequent primary or secondary lesions of the liver. Isolated hepatic involvement by tuberculosis can especially be challenging to diagnose on imaging alone due its largely non-specific imaging features. Nevertheless, in endemic countries and in appropriate clinical settings an atypical imaging pattern of a hepatic lesion should prompt the radiologist to consider hepatic tuberculosis as one of the differential considerations. Although image-guided biopsy is usually required for a confirmatory diagnosis the presence of calcifications and the concurrent involvement of extrahepatic sites (spleen, lungs and nodes) should prompt the possibility of hepatobiliary tuberculosis.
